# Psychological mechanisms of behavioral change: Trajectories of self-efficacy and motivation among Tunisian health science students using a mobile application to quit smoking - A prospective observational study

**DOI:** 10.1371/journal.pgph.0006081

**Published:** 2026-03-04

**Authors:** Selma Gallas, Houyem Said Latiri

**Affiliations:** 1 Medical Intensive Care Unit, Farhat Hached University Hospital, University of Sousse, Faculty of Medicine of Sousse, Sousse, Tunisia; 2 Department of Preventive and Community Medicine, Sahloul University Hospital, University of Sousse, Faculty of Medicine of Sousse, Sousse, Tunisia; Cancer Institute Women's India Association, INDIA

## Abstract

Tobacco use remains a leading preventable cause of morbidity and mortality worldwide, with high prevalence among Tunisian health sciences students. Psychological determinants such as motivation and self-efficacy are key predictors of smoking cessation success. Mobile health (mHealth) interventions offer scalable support, yet their influence on these psychological mechanisms is underexplored in specific contexts. This study aimed to evaluate changes in motivation and self-efficacy to quit smoking among health sciences students using a culturally adapted mobile app in Tunisian dialect, and to examine associations with smoking behavior. A prospective observational study was conducted in 2023 involving 192 daily smoking first-year health sciences students in Sousse, Tunisia. Participants used the app for one month and completed baseline and follow-up assessments of motivation (Q-MAT) and smoking self-efficacy scales, nicotine dependence and smoking behavior were recorded. Paired t-tests and regression analyses assessed changes and predictors of psychological outcomes. Among 107 participants completing follow-up, motivation scores significantly increased from 5.6 ± 4.23 to 9.7 ± 3.26 (p < 0.001), with a redistribution toward higher motivation levels. Although overall self-efficacy scores remained stable, the proportion of participants with high self-efficacy increased significantly (p < 0.001).. Changes in motivation and self-efficacy were positively correlated (r = 0.76, p = 0.001) and associated with reductions in smoking behavior. The use of a culturally appropriate mobile application increases motivation and self-efficacy to quit smoking among Tunisian health science students, thereby promoting smoking reduction. Mobile interventions targeting these psychological mechanisms can complement traditional cessation strategies. Conventional approaches, including face-to-face counseling, pharmacotherapy, group therapy, and telephone support, are effective but often difficult to implement widely in resource-limited settings. In Tunisia and similar contexts, barriers such as limited access to qualified professionals, high costs, geographical constraints, and stigma may restrict their use. Mobile health interventions therefore represent an accessible and cost-effective complementary solution.

## Background

Smoking remains one of the leading preventable causes of morbidity and mortality worldwide, responsible for more than 8 million deaths annually and generating a significant economic burden on health systems [1].

The prevalence of smoking among Tunisian students shows a worrying upward trend. In 2020, the prevalence of smoking among medical students aged18–25 Years in Monastir was 10.7% [2]. At the regional level, Tunisia has particularly high rates, with a smoking prevalence of over 40% among male university students and 3.4% among female students, placing it among the highest in the Arab region [3].

Health sciences students were selected due to both developmental and professional factors. As young adults transitioning to university, they face increased independence, peer influence, and academic stress factors associated with tobacco initiation and continuation [4]. Despite their future role as healthcare providers, only 4.5% of final-year medical students feel prepared to deliver cessation counseling, and 65.7% would not advise quitting in asymptomatic patients [5]. In Tunisia, smoking prevalence reaches 38.4% among male and 3.4% among female university students, and among health sciences students is significantly associated with male sex (OR=6.929), Pharmacy faculty (OR=3.081), and academic difficulties (OR=1.854) [3]. Given their high mobile technology use, mHealth interventions are particularly suitable for this vulnerable and strategically important group.

To understand and support smoking cessation, it is crucial to consider psychological determinants such as motivation and self-efficacy. According to Bandura’s social cognitive theory, self-efficacy is defined as an individual’s confidence in their ability to resist consumption in high-risk situations [6–8]. Motivation to quit, conceptualized as perceived importance and determination to succeed in an attempt, is also a recognized predictor of successful cessation [9,10].

However, access to traditional interventions is often limited by geographical, financial, and social constraints, particularly among young adults and students. Mobile health (mHealth) technologies offer a scalable and accessible solution for delivering interventions to support smoking behavior change [11,12]. Nevertheless, few studies have explored how these tools specifically influence the key psychological mechanisms of self-efficacy and motivation.

### Study objectives

To evaluate the temporal evolution of motivation and self-efficacy to quit smoking among health science students using a culturally adapted application in the Tunisian dialect.

## Methods

### Study design and setting

This prospective observational study was conducted in 2023 across several health sciences institutions in Sousse, Tunisia, including public and private nursing schools and the Faculty of Medicine. The study adhered to standard guidelines for observational research and was approved by the Ethics Committee of Tunisia (registration number HS12–2022).

### Participant recruitment and eligibility

Students were recruited through institutional announcements in March 2023. Eligible participants met the following criteria: (1) age ≥ 18 years; (2) daily smokers for at least one year; (3) first-year health sciences students (medicine or nursing); (4) ownership of an Android smartphone compatible with the app in Tunisian dialect; (5) willingness to use the assigned mobile app; and (6) provision of informed consent online.

Exclusion criteria included: (1) severe psychiatric or neurological disorders; (2) current participation in medically supervised smoking cessation programs; (3) concurrent use of other smoking cessation apps; and (4) refusal to complete required assessments.

### Sample size

Sample size calculation was based on the accessible population and prior data from Lüscher et al., 2019 [13]. To detect an effect with 80% power at a 0.05 significance level, at least 86 participants were required. Anticipating a 44% attrition rate, 192 participants were recruited. After one month, 107 participants completed the study, exceeding the minimum required sample. Completion required using the app at least once per week and completing all questionnaires.

### Design of the mHealth intervention: Theoretical framework and essential components

The mHealth intervention was developed specifically for this study rather than being adapted from existing applications, drawing on social cognitive theory (Bandura) and the transtheoretical model (stages of change). Development followed a three-phase iterative process, combining evidence-based strategies with features tailored to the culture and relevant to Tunisian health science students.

Phase 1_Formative research: Focus groups composed of 20 health science students explored culturally important motivators, common barriers, and preferred communication styles. The findings from these discussions informed the design of messages, goal-setting tools, and engagement strategies.
Phase 2_Intervention design: An interdisciplinary team, consisting of a psychologist specializing in behavioral health, a smoking cessation specialist, and a mobile app developer, developed the content and features of the intervention.
Phase 3_ Pilot testing: Usability testing with 15 students enabled iterative improvements based on user feedback, engagement metrics, and cultural fit assessment.

The application was culturally adapted to the Tunisian context by translating it into Tunisian dialect/French, following the various stages of cross-cultural translation. Participants were encouraged to use the application daily, with a recommended frequency of use.

And the fidelity of the intervention was monitored through app usage data (connection frequency, features consulted).

### Assessment and follow-up

Participants were assessed at baseline (Day 0) and after four weeks of app use, based on [14]. Outcomes included smoking-related self-efficacy and motivation to quit.

### Primary outcomes

**Sociodemographic Factors:** Collected at baseline, including age, sex, residence, origin, family situation, and socioeconomic status.

**Nicotine Dependence:** Assessed using the six-point Fagerström Test. Participants were categorized as low (0–4), moderate (5–7), or high dependence (8–10).

**Previous Quit Attempts:** Participants reported the number of serious quit attempts in the past 12 months.

**Smoking Self-Efficacy:** Measured using a 9-item scale assessing confidence to resist smoking in high-risk situations (Likert 1–5). Total scores range from 9 to 45, with higher scores indicating greater self-efficacy.

Two subscales were assessed: intrinsic self-efficacy, reflecting confidence in managing internal challenges such as nicotine cravings, withdrawal symptoms, and emotional triggers; and extrinsic self-efficacy, reflecting confidence in handling external challenges such as social pressure, exposure to smoking environments, and academic or work-related stressors. Both dimensions are critical predictors of successful long-term cessation.

**Motivation to Quit (Q-MAT):** A validated questionnaire assessing intrinsic and extrinsic motivation, self-determination, and confidence in quitting success. Items scored on a 7-point Likert scale; higher scores reflect stronger motivation.

### Statistical analysis

Data analysis was performed using a per-protocol approach, including only participants who completed all follow-up assessments (n = 192).

Data were analyzed using SPSS. Descriptive statistics summarized participant characteristics, smoking habits, and previous quit attempts. Paired t-tests assessed changes in self-efficacy and motivation between baseline and follow-up. Linear regression models examined associations between changes in self-efficacy and motivation and demographic factors (age, sex, nicotine dependence). Statistical significance was set at p < 0.05, and 95% confidence intervals were reported.

The assumptions of linear regression were systematically verified before the analysis:

1. Normality of residuals: verified by the Shapiro-Wilk test
2. Homoscedasticity: verified by the Breusch-Pagan test and inspection of standardized residual plots.
3. Independence of residuals: assessed using the Durbin-Watson test.
4. Absence of multicollinearity: confirmed by calculating VIF (Variance Inflation Factor < 5 for all variables).
5. Linearity of relationships: verified by examining scatter plots.

Note: Due to high multicollinearity between self-efficacy, resistance to external stimuli, and resistance to internal stimuli (r = 0.89-0.99), we retained only self-efficacy as a representative of this psychological dimension to avoid statistical issues.

### Ethical considerations

The study was approved by the Ethics Committee of CHU Farhat Hached Sousse (HS12–2022). Institutional permissions were obtained prior to data collection. Participants were informed about the study objectives and conditions through an online consent form. Confidentiality and anonymity of data were maintained throughout.

## Results

### Flowchart des participants

**Fig 1:**
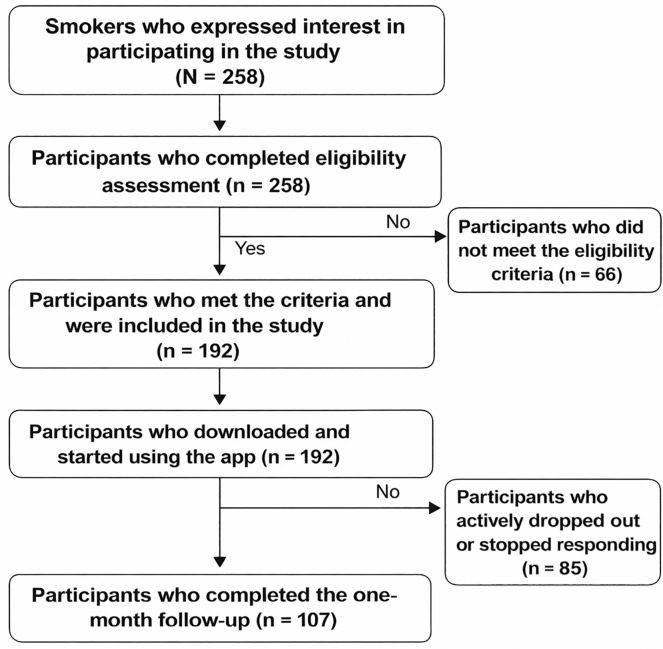
Overview of participant numbers from expression of interest to study completion.

Participant flow chart. Of 258 interested smokers, 192 met the eligibility criteria and used the app. At the one-month follow-up, 107 participants completed the final assessment, while 85 withdrew or stopped responding (Fig 1).

### Basic characteristics

Among the 192 participants, the average age was 18.1 ± 1.2 years, with a sex ratio of 1.82 (64.6% male). The majority lived in urban areas (65.6%), had living parents (73.4%), and had a fairly satisfactory socioeconomic status (62%). Most lived in university housing (58.6%) or with their families (27.1%) (Table 1).

**Table 1:** Baseline characteristics of health sciences students’ smokers (n = 192).

	n	%	M ± SD
**Age (years)**			18.1 ± 1.2
Gender			
Female	68	35.4	
Male	124	64.6	
**Origin**			
Urban	126	65.6	
Rural	66	34.4	
**Family situation**			
Parents living together	141	73.4	
Divorced parents	31	16.2	
Orphan	20	10.4	
**Socioeconomic status**			
Unsatisfactory	37	19.3	
fairly satisfactory	119	62.0	
Satisfactory	36	18.7	
**Place of residence**			
With family	52	27.1	
In a university residence	113	58.6	
Shared accommodation	17	8.8	
Individual rental	8	4.4	
With a relative	2	1.1	
**Age at onset of smoking**			17.70 ± 1.2
**Attempts to quit (yes)**	58	30.2	
**Maximum duration of abstinence**		59.2 ± 31.1 hours	

Mean daily cigarette consumption remained stable between D0 and M1 (14.3 ± 8.2 vs 13.2 ± 8.1 cigarettes/day, p = 0.541), with no significant difference. Similarly, Fagerström test scores showed no significant change (2.3 ± 1.2 vs 2.4 ± 1.1, p = 0.223). The distribution of dependence levels shifted slightly, with moderate dependence increasing from 39.1% to 51.4%, while low and high dependence decreased from 19.3% to 14.0% and from 41.6% to 34.6%, respectively (Table 2).

**Table 2:** Smoking characteristics and levels of dependence at D0 and M1.

	D0 (n = 192)			M1 (n = 107)			
	N	%	M ± SD	N	%	M ± SD	p
**Cigarettes/day**			14.3 ± 8,2			13,2 ± 8,1	0.541
**Fagerström test**			2.3 ± 1.2			2.4 ± 1.1	0.223
Low dependence	37	19.3		15	14.0		
Moderate dependence	75	39.1		55	51.4		
High dependence	80	41.6		37	34.6		

Of the 192 students recruited, 107 completed the study after using the app for one month.

#### Motivation.

The average motivation of participants increased from 5.6 ± 4.23 to 9.7 ± 3.26 (p < 0.001), with a significant increase in the number of participants with high motivation (p < 0.001). The percentage of participants with low motivation fell from 59.4% to 14.9% (Table 3).

**Table 3:** Variations in Q-MAT scores and motivation levels between J0 and M1.

Variables	D0 (n = 192)	M1 (n = 107)	p-value
**Q-MAT score, M ± SD**	5.6 ± 4.23	9.7 ± 3.26	< 0.001
**Q-MAT categories, n (%)**			
**Low motivation**	114 (59.4%)	16(14.9%)	< 0.001
**Moderate motivation**	53(27.6%)	67(62.7%)	
**High motivation**	25 (13.0%)	24 (22.4%)	

#### Self-efficacy.

Although the average self-efficacy score did not change significantly (24.78 ± 4.86 to 24.69 ± 5.53), a higher proportion of participants showed high self-efficacy (p < 0.001). Resistance to internal and external stimuli also showed significant improvements (p < 0.001 and p = 0.002) (Table 4).

**Table 4:** Variation in smoking-related indicators between D0 and M1.

	D0 (n = 192)			M1 (n = 107)			
	n	%	M ± SD	n	%	M ± SD	p
**Smoking self-efficacy scale**			24.78 ± 4.86			24.69 ± 5.53	<0.001
Low self-efficacy	97	50.5		63	58.9		
Moderate self-efficacy	76	39.6		20	18.7		
High self-efficacy	19	9.9		24	22.4		
External stimuli			3.9 ± 3.34			3.6 ± 3.31	<0.001
Low resistance to external stimuli	143	74.5		54	50.5		
High resistance to external stimuli	49	25.5		53	49.5		
Internal stimuli		9.91 ± 8.28				8.82 ± 8.54	0.002
Low resistance to internal stimuli	93	48.4		27	25.3		
High resistance to internal stimuli	99	51.6		80	74.7		

*Tests utilisés: test t de Student pour données continues — Khi² pour proportions. Test utilisé: χ² de Pearson.

#### Correlations.

A strong correlation was observed between changes in motivation and self-efficacy (r = 0.76) (Table 5).

**Table 5:** Correlation matrix between sociodemographic variables, smoking indicators, and psychosocial dimensions.

Variables	Age	Economic status	Age at onset of smoking	Cigarettes/day	Attempts to quit	Maximum duration of abstinence	Fagerström	Overall motivation	External stimuli	Internal stimuli	Self-efficacy
**Age**	**1.00**	**0.17**	**0.18**	**0.07**	**–**	**0.15**	**-0.18**	**0.03**	**0.03**	**0.03**	**0.06**
**Economic status**	**0.17**	**1.00**	**0.24**	**-0.19**	**–**	**0.38**	**-0.45**	**0.56**	**0.35**	**0.32**	**0.32**
**Age at onset of smoking**	**0.18**	**0.24**	**1.00**	**0.23**	**–**	**0.26**	**-0.12**	**0.09**	**-0.09**	**-0.05**	**-0.05**
**Cigarettes/day**	**0.07**	**-0.19**	**0.23**	**1.00**	**–**	**-0.18**	**-0.08**	**-0.03**	**-0.08**	**-0.12**	**-0.12**
**Attempts to quit**	**–**	**–**	**–**	**–**	**1.00**	**–**	**–**	**–**	**–**	**–**	**–**
**Maximum duration of abstinence**	**0.15**	**0.38**	**0.26**	**-0.18**	**–**	**1.00**	**-0.21**	**0.25**	**0.17**	**0.07**	**0.01**
**Fagerström**	**-0.18**	**-0.45**	**-0.12**	**-0.08**	**–**	**-0.21**	**1.00**	**-0.59**	**-0.57**	**-0.54**	**-0.54**
**Overall motivation**	**0.03**	**0.56**	**0.09**	**-0.03**	**–**	**0.25**	**-0.59**	**1.00**	**0.77**	**0.78**	**0.76**
**External stimuli**	**0.03**	**0.35**	**-0.09**	**-0.08**	**–**	**0.17**	**-0.57**	**0.77**	**1.00**	**0.88**	**0.89**
**Internal stimuli**	**0.03**	**0.32**	**-0.05**	**-0.12**	**–**	**0.07**	**-0.54**	**0.78**	**0.88**	**1.00**	**0.99**
**Self-efficacy**	**0.06**	**0.32**	**-0.05**	**-0.12**	**–**	**0.01**	**-0.54**	**0.76**	**0.89**	**0.99**	**1.00**
	**Strong positive correlation r ≥ 0.7**						**Strong negative correlation │r│ ≥ 0.7**				
	**Moderate positive correlation 0.5 ≤ r < 0.7**						**Moderate negative correlation 0.5 ≤ │r│ < 0.7**				
	**Weak positive correlation 0.3 ≤ r < 0.5**						**Weak negative correlation 0.3 ≤ │r│ < 0.5**				
	**Very weak positive correlation 0.1 ≤ r < 0.3**						**Very weak negative correlation 0.1 ≤ │r│ < 0.3**				

The multiple linear regression model was statistically significant (F(5, 183) = 11.42, p < 0.001) and explained 23.8% of the variance in smoking cessation motivation (R² = 0.238; adjusted R² = 0.217). Socioeconomic status was the only variable significantly associated with motivation to quit smoking (standardized β = 0.507, p < 0.001). Higher socioeconomic status was associated with higher motivation scores.

No significant associations were observed for nicotine dependence as measured by the Fagerström score, self-efficacy, age, or number of cigarettes smoked per day (p > 0.05 for all). The confidence intervals for these predictors included zero, indicating no statistically significant effect in this model (Table 6).

**Table 6:** Results of multiple linear regression predicting smoking cessation motivation.

Predictor Variables	Unstandardized β	SE	Standardized β	t	p	95% CI
(Constant)	8.202	3.809	--	2.153	0.033	[0.69, 15.72]
Socioeconomic status	2.826	0.415	0.507	6.811	< 0.001	[2.01, 3.65]
Fagerström score	-0.042	0.225	-0.016	-0.188	0.851	[-0.49, 0.40]
Self-efficacy	-0.012	0.020	-0.049	-0.626	0.532	[-0.05, 0.03]
Age	-0.212	0.187	-0.075	1.133	0.259	[-0.58, 0.16]
Cigarettes per day	0.036	0.028	0.088	1.288	0.199	[-0.02, 0.09]

**Model Statistics:**

R² = 0.238; Adjusted R² = 0.217

F(5, 183) = 11.42; p < 0.001

n = 189 participants

**Note:** Dependent variable = Q-MAT motivation score at M1. β = regression coefficient; SE = standard error; CI = confidence interval. The model explains 23.8% of the variance in motivation. Socioeconomic status was the only significant predictor (p < 0.001).

## Discussion

The high proportion of participants who had never tried to quit smoking before (30.2%) suggests that this mobile app may have attracted first-time quitters, likely due to its accessibility and low barrier to entry. This result highlights the potential of mHealth interventions to reach populations that do not use traditional smoking cessation devices.

Recent data shows that about 40% of Chinese apps target motivation, underscoring its importance in digital interventions [15]. The short average duration of abstinence (59.2 ± 31.1 hours) aligns with existing literature, which indicates that attempts without support often fail within the first 72 hours. Borrelli et al. (2024) confirm that interventions addressing mood and motivation during this critical phase can extend abstinence [16].

### Motivation: Early and sustained improvement

A key finding of this study is the significant increase in motivation observed from the first month of the intervention, which is clinically relevant given that early improvements in motivation have been associated with longer-term smoking cessation outcomes. This increase likely reflects the effectiveness of the initial components of the intervention, including testimonials from healthcare professionals, visual representations of health risks, and personalized motivational messages. Consistent with previous research, this finding suggests that digital interventions may be particularly effective in supporting individuals during the transition from contemplation to action [17].

What stands out in this study is the high level of motivation maintained during the follow-up period. Many smoking cessation interventions report a decline in motivation over time as initial enthusiasm fades and the challenges of behavior change become more apparent [18,19]. Our results suggest that well-designed mobile apps can not only elicit rapid engagement but also sustain motivation over the medium term. We operationalized medium-term motivation as sustained motivation assessed one month after the intervention, recognizing that the initial 30-day period represents a critical window of vulnerability to relapse. This timeframe aligns with behavioral science literature, which shows that initial habit disruption and self-efficacy consolidation typically occur within the first 3–6 weeks of quit attempts. This finding is consistent with Rajani et al. (2021), who observed significant increases in motivation among adult smokers using gamified apps over a four-week period [14].

### Self-efficacy: Gradual development

Unlike motivation, self-efficacy developed more gradually. Participants seemed to gain confidence in their ability to resist cravings and navigate risky situations through successful withdrawal experiences, thereby strengthening their behavioral skills. This gradual development of self-efficacy aligns with Bandura’s social cognitive theory, which posits that self-efficacy is built through mastery of concrete experiences [6]. Recent meta-analyses confirm that both motivation and self-efficacy are among the best predictors of successful withdrawal attempts [20,21].

### The relationship between motivation and self-efficacy

Correlation analysis revealed several important patterns regarding the determinants of smoking behavior. The moderate to strong negative correlations observed between nicotine dependence (measured by the Fagerström scale) and psychosocial variables (overall motivation r = –0.59, self-efficacy r = –0.54) confirm that dependence is a significant barrier to behavior change, which is consistent with the findings of Kim et al. and Cupertino et al., who highlighted the predictive role of self-efficacy in successful cessation attempts [22,23].

The high correlations between motivation, self-efficacy, and other psychosocial factors (r = 0.77 to 0.89) suggest a strong interdependence among these variables. This synergy justifies the use of multi-component approaches, which have been shown to be more effective, particularly when personalized mobile interventions target multiple motivational levers in young smokers [24,25].

Interestingly, weak correlations between behavioral variables (e.g., cigarettes/day, age of onset) and psychosocial factors (|r| < 0.23) suggest that smoking intensity does not necessarily correlate with a smoker’s psychosocial profile. This finding highlights the importance of multidimensional assessments when designing smoking cessation interventions, especially for younger populations [26].

### Temporal dynamics: Motivation and self-efficacy

The temporal dynamics observed in this study where motivation increases rapidly followed by a gradual strengthening of self-efficacy are consistent with theoretical models in which motivation facilitates the initiation of change, while self-efficacy supports maintenance and relapse prevention [16]. Similar patterns have been reported in studies targeting young adult and university populations, suggesting that early engagement through motivational strategies is critical for this demographic. Traditional cessation programs often face barriers in this group, including limited use of pharmacotherapy and counseling, time constraints, discomfort with clinical settings, stigma, and, for health sciences students, concerns about confidentiality and professional identity. Our findings indicate that mHealth interventions can overcome these limitations by providing anonymous, accessible, and personalized support that enhances motivation quickly and builds self-efficacy over the medium term, offering a practical approach where conventional programs are underutilized or less effective [21].

The strong correlation (r = 0.76) between motivation and self-efficacy suggests a reciprocal relationship: increased motivation leads to greater engagement in skills-building modules, while progressive successes in self-efficacy further reinforce motivation [14].

Our observed attrition rate of 44% for the mobile app is similar to patterns reported in the recent systematic review by Fang and al, 2023, which noted that many eHealth smoking cessation interventions especially mobile health approaches exhibit decreasing user engagement over time, even when short-term effectiveness is demonstrated. Although Fang et al. did not quantify average dropout rates across all included studies, they highlighted that intervention effects, including sustained abstinence and engagement, tend to diminish as follow-up length increases, reflecting challenges in maintaining long-term participation in digital cessation programs. This consistency suggests that high attrition is a common issue in mHealth smoking cessation interventions and underscores the importance of strategies to improve sustained engagement [20].

### Theoretical and clinical implications

This study supports the theoretical model that motivation and self-efficacy are key cognitive and motivational drivers for quitting. Clinically, it demonstrates that culturally adapted mobile applications can complement traditional support, particularly in settings with limited access to face-to-face counseling services. The results indicate that the gradual reinforcement of self-efficacy, combined with an early motivational boost, is a central mechanism for facilitating progression toward action and maintenance.

### Limitations and implications

Self-reported measures may be influenced by social desirability or recall bias. Attrition could introduce bias if participants who dropped out differed from those who did not. Integrating mobile tools into health student training could strengthen motivational counseling skills and support smoking cessation, particularly in settings with limited access to in-person support.

## Conclusion

This study shows that the use of a culturally adapted mobile application increases the motivation and self-efficacy of health science students to quit smoking. These results highlight the potential of digital interventions as a complement to traditional cessation methods, particularly in resource-limited settings. Future research should explore the long-term effectiveness and integration of these tools into public health policies in Tunisia.
